# CRISPR/Cas9 -mediated gene knockout of *Anopheles gambiae FREP1* suppresses malaria parasite infection

**DOI:** 10.1371/journal.ppat.1006898

**Published:** 2018-03-08

**Authors:** Yuemei Dong, Maria L. Simões, Eric Marois, George Dimopoulos

**Affiliations:** 1 W. Harry Feinstone Department of Molecular Microbiology and Immunology, Bloomberg School of Public Health, Malaria Research Institute, Johns Hopkins University, Baltimore, Maryland, United States of America; 2 Inserm, CNRS, Université de Strasbourg, Strasbourg, France; Institut Pasteur, FRANCE

## Abstract

*Plasmodium* relies on numerous agonists during its journey through the mosquito vector, and these agonists represent potent targets for transmission-blocking by either inhibiting or interfering with them pre- or post-transcriptionally. The recently developed CRISPR/Cas9-based genome editing tools for *Anopheles* mosquitoes provide new and promising opportunities for the study of agonist function and for developing malaria control strategies through gene deletion to achieve complete agonist inactivation. Here we have established a modified CRISPR/Cas9 gene editing procedure for the malaria vector *Anopheles gambiae*, and studied the effect of inactivating the fibrinogen-related protein 1 (*FREP1*) gene on the mosquito’s susceptibility to *Plasmodium* and on mosquito fitness. *FREP*1 knockout mutants developed into adult mosquitoes that showed profound suppression of infection with both human and rodent malaria parasites at the oocyst and sporozoite stages. *FREP1* inactivation, however, resulted in fitness costs including a significantly lower blood-feeding propensity, fecundity and egg hatching rate, a retarded pupation time, and reduced longevity after a blood meal.

## Author summary

The causative agent of malaria, *Plasmodium*, has to complete a complex infection cycle in the *Anopheles gambiae* mosquito vector in order to reach the salivary gland from where it can be transmitted to a human host. The parasite’s development in the mosquito relies on numerous host factors (agonists), and their inhibition or inactivation can thereby result in suppression of infection and consequently malaria transmission. The recently developed CRISPR/Cas9-based genome editing tools for *Anopheles* mosquitoes provide new and promising opportunities to delete (inactivate) *Plasmodium* agonists to better understand their function and for blocking malaria transmission. Here we have established a modified CRISPR/Cas9 genome editing technique for malaria vector *A*. *gambiae* mosquitoes. Through this approach we have inactivated the fibrinogen-related protein 1 (FREP1) gene, via CRISPR/Cas9 genome editing, and the impact of this manipulation on the mosquito’s susceptibility to *Plasmodium* and on mosquito fitness. *FREP*1 knockout mutants showed a profound suppression of infection with both human and rodent malaria parasites, while it also resulted in fitness costs: a significantly lower blood-feeding propensity, fecundity and egg hatching rate, and a retarded larval development and pupation time, and reduced longevity after a blood meal.

## Introduction

Malaria remains one of the most devastating infectious diseases, killing nearly half a million people annually [1]. For successful transmission, the malaria parasite has to complete a complex journey in the mosquito vector that involves intimate molecular interactions with the vector’s midgut, hemolymph, and salivary glands [2]. These crucial *Anopheles-Plasmodium* interactions represent targets for transmission blocking by inhibiting parasite agonists (or host factors) that are required for infection [3–6]. An obvious advantage of studying *Plasmodium* agonists for malaria control is that they can be targeted through multiple means, such as agonist gene deletion, RNAi-mediated gene silencing, or interference through antibodies or small molecules [5, 7–8]. However, the exploration of mosquito *Plasmodium* agonists for the development of malaria control strategies based on parasite suppression has lagged behind other approaches because of the lack of effective gene editing tools for *Anopheles* mosquitoes. The recently developed CRISPR/Cas9-based genome editing methodology and gene-drive systems for *Anopheles* mosquitoes provide new and promising tools for the study of *Plasmodium* host factors, with regard to their biology and potential for the development of transmission-blocking strategies.

Type II clustered regularly interspaced short palindromic repeats (CRISPR) bacterial immune systems, together with CRISPR-associated protein 9 (Cas9), have been developed into a powerful tool for genome engineering, and CRISPR/Cas9-based gene editing has been widely explored for biomedical research and therapeutics. This technology has recently also been applied to genome editing in *Aedes aegypti*, *Anopheles stephensi*, and *Culex* mosquitoes [9–14]. Furthermore, Kistler and colleagues [11] have established a comprehensive protocol for CRISPR/Cas9 gene editing in *Aedes* mosquitoes through embryonic delivery of *in vitro*-synthesized guide RNA (sgRNA) and recombinant Cas9 protein. CRISPR/Cas9 mediated somatic disruption of a male-determining gene in *Aedes* mosquitoes has produced males with feminized genitalia [12]. Cas9-mediated gene drive technology has proven promising for population modification of both Asian and African malaria vector mosquitoes, *A*. *stephensi* and *A*. *gambiae*, respectively [15–17].

Here we investigated the effect of fibrinogen-related protein 1 (FREP1) gene inactivation, obtained by CRISPR/Cas9 genome editing, on both susceptibility to *Plasmodium* and mosquito fitness. FREP1 belongs to the fibrinogen-related protein family (FREP, also known as fibrinogen domain immunolectin [FBN]), representing the largest immune- and pattern recognition gene family in *A*. *gambiae*, with 59 putative members. We have previously shown that several members of the FREP/FBN family are infection-responsive upon challenge with bacteria or *Plasmodium*, and FBN8 (FREP57), FBN9 (FREP13), and FBN39 (FREP40) are involved in anti-*Plasmodium* defense [18–19]. Transgenic overexpression of *FBN9* (*FREP13*) in *A*. *gambiae* mosquitoes results in an elevated resistance to infections with bacteria and the rodent malaria parasite *P*. *berghei* [20].

Whereas several FREP members are *Plasmodium* antagonists, FREP1 functions as broad-spectrum *Plasmodium* agonist (host factor) [21–23]. FREP1 plays a potent role in the *Plasmodium* ookinete’s invasion of the mosquito midgut epithelium [21]. RNAi-based studies have shown that gene silencing of *FREP*1 results in a suppression of *P*. *falciparum* development in the midgut tissue [21]. Polyclonal anti-FREP1 antibodies can block infection of *Anopheles* with *P*. *falciparum*, *P*. *vivax*, and *P*. *berghei*, suggesting that FREP1 might be a promising transmission-blocking vaccine (TBV) target [23]. While such a TBV has already been developed, here we have taken a different interference approach, using *FREP1* gene disruption to investigate its biological role as a host factor and its potential as a transmission-blocking target for the development of a novel malaria control strategy.

To generate CRISPR/Cas9-based *FREP1* knockout *A*. *gambiae* mosquitoes, we established a modified CRISPR/Cas9 gene editing method based on crossing a guide RNA (gRNA)-expressing transgenic line with a transgenic line expressing Cas9 under the control of the germline-specific *Vasa2* promoter [24]. Mosquitoes carrying the desired knockout mutation were isolated in the progeny. The resulting *FREP1* knockout mutant mosquitoes showed a significant decrease in their permissiveness to *P*. *falciparum* and *P*. *berghei* infection. However, the mutants also exhibited a significant fitness cost in terms of blood-feeding propensity, longevity after blood meal, fecundity, and egg hatching rates, but no impact on the adult mosquito size or on the life-span of mosquitoes maintained on a 10% sugar solution.

## Results

### Generation and characterization of *FREP1* knockout mutants

In our preliminary attempts to generate CRISPR/Cas9 knockout null mutants we tried the method established for *Aedes* and *Anopheles stephensi* mosquitoes [11, 15] by co-injecting synthesized gRNAs (sgRNAs) and Cas9 protein into the embryos of *A*. *gambiae* G3 mosquitoes. The mosquito embryo microinjection was carried out according to established protocols [25–28]. In total, approximately 10% of the eggs hatched from 1000 micro-injected eggs, and the emerged G_0_ adults were allowed to outcross with wild type mosquitoes to produce G_1_ eggs followed by screening for germline transmission of stable mutations, but not a single mutant was identified in the survivors’ progeny.

**Fig 1:**
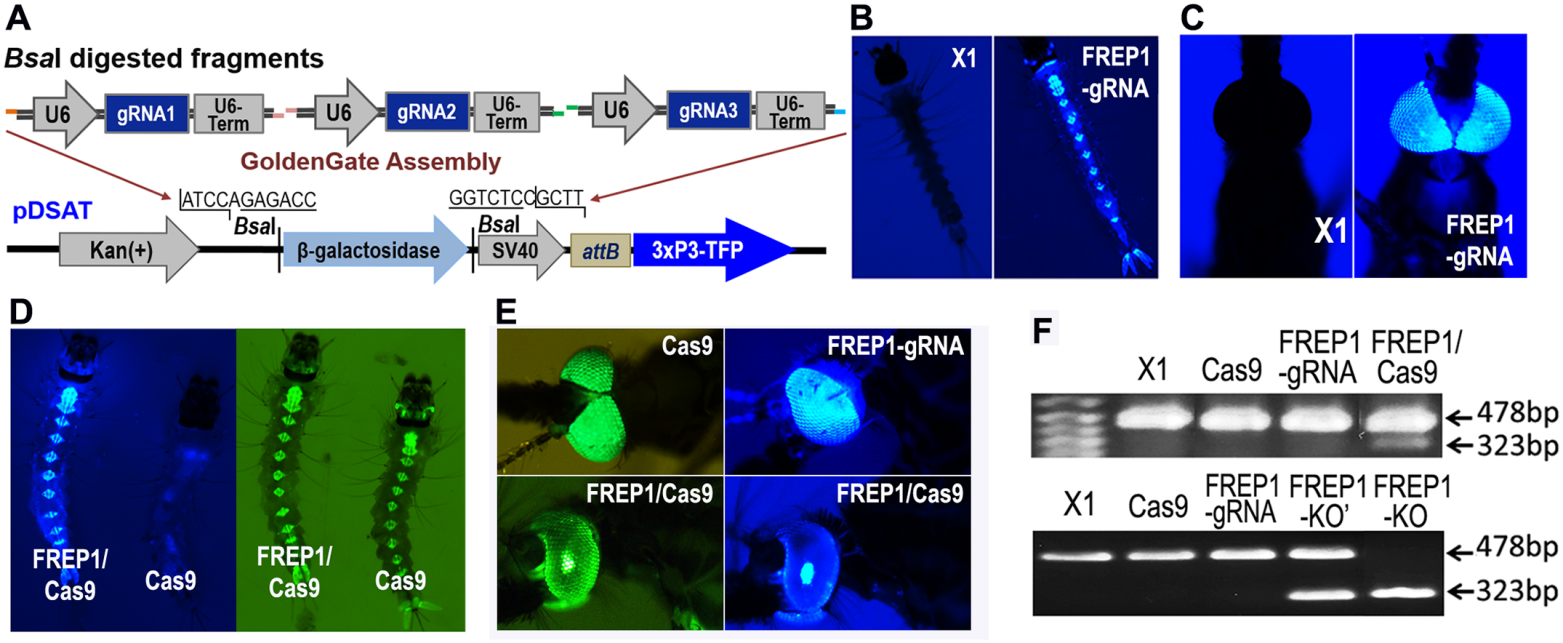
Generation of CRISPR/Cas9-mediated *FREP1* gene knockout mutants. (**A**) Schematic representation of the pDSAT-FREP1-gRNA_3_ (with blue fluorescence eye reporter gene) transformation plasmid used for the germline transformation of the *A*. *gambiae* X1 docking line. Three gRNA target DNA sequences of FREP1 were first independently cloned into gRNA expression template vectors and assembled into a pDSAT vector via the Golden Gate cloning technique using *Bsa*I sites. (**B, C)** Generation of *FREP1*-*gRNA*-expressing (FREP1-gRNA) transgenic line. Fluorescent images of larvae of FREP1-gRNA (blue) transgenic line, along with the wt control (X1, non-fluorescent) strain (**B**). Fluorescent images of adult transgenic FREP1-gRNA mosquito and wt control (**C**). (**D, E**) FREP1-gRNA virgin females were crossed with *Vasa-Cas9* (Cas9, YFP, yellow/green fluorescence) males to generate the transheterozygotes (FREP1/Cas9) for producing *FREP1* gene knockouts. The transheterozygous progeny has both blue and green fluorescence in larval (**D**) and adult eyes (**E**), whereas progeny that did not inherit FREP1-gRNA has only the green fluorescent eye marker. (**F**) PCR validation of *FREP1* gene disruption (sequences available in SI S1 File). 478 bp PCR products from X1, Cas9, FREP1-gRNA, and FREP1/Cas9 transheterozygotes were sequenced (upper panel). PCR showed that the heterozygous *FREP1* mutants have both wild type band (478 bp) and the lower band (323 bp) with a 155 bp gene deletion (FREP1-KO’). Homozygous deletion mutant (FREP1-KO) showed only one lower band (SI S1 File).

We then proceeded with the development of a modified method to generate CRISPR/Cas9-induced knockout mutant in *A*. *gambiae* mosquitoes through combination and modification of established approaches [16, 28]. We first created a transgenic *A*. *gambiae* line expressing three gRNAs targeting the *FREP1* gene (S1 Fig). All three *FREP1*-targeting gRNAs were driven by the *A*. *gambiae* U6 snRNA polymerase III promoter (AGAP013557). The specific sequences of individual gRNAs were synthesized as short DNA linkers (S1 Table) and separately cloned into plasmid modules carrying the U6 promoter sequence followed by a gRNA-expression template, and assembled by a Golden Gate cloning reaction into the pDSAT transgenesis vector [28] containing the 3xP3-TFP reporter (Figs 1A and S1). Upon microinjection into embryos of the *A*. *gambiae* X1 docking line [28] and backcrossing of injected survivors to the parental X1 line, transgenic progeny showing stable blue fluorescence in the larvae or adult eyes were isolated for further studies (Fig 1B and 1C). After amplifying the *FREP1*-*gRNA*-expressing (FREP1-gRNA) transgenic population, a subpopulation enriched with homozygous transgenic mosquitoes was crossed with the *Vasa-Cas9* strain [15–16] to generate germ-line gene-knockout mutants. Males from this cross carrying both transgenes were selected by their fluorescent markers (Fig 1D and 1E), and outcrossed to wild type (wt) virgin females (S1 Fig). Only the virgin female progeny from this cross (being potential heterozygous mutants) was crossed with wt males. These females were blood-fed and isolated in individual containers to lay eggs. The mutation region from the mother was assessed by PCR with flanking primers (S1 Table) and the mutation/deletion was confirmed by PCR first (Fig 1F) followed by sequencing (SI S1 File). In the progeny from these founder females, PCR on leg DNA was used to screen individual mutant mosquitoes, and all the mutant mosquitoes with the same heterozygous mutation genotype as revealed by PCR and sequencing were pooled. To enrich for homozygous mutants, the whole process was repeated in the next generation to generate homozygous deletion mutants (Fig 1F). The reading frames of *FREP1 gene* were shifted and no FREP1 protein was produced in *FREP1*-KOs (SI S1 File).

### Impact of *FREP1* knockout on susceptibility to the human malaria parasite *P*. *falciparum*

**Fig 2:**
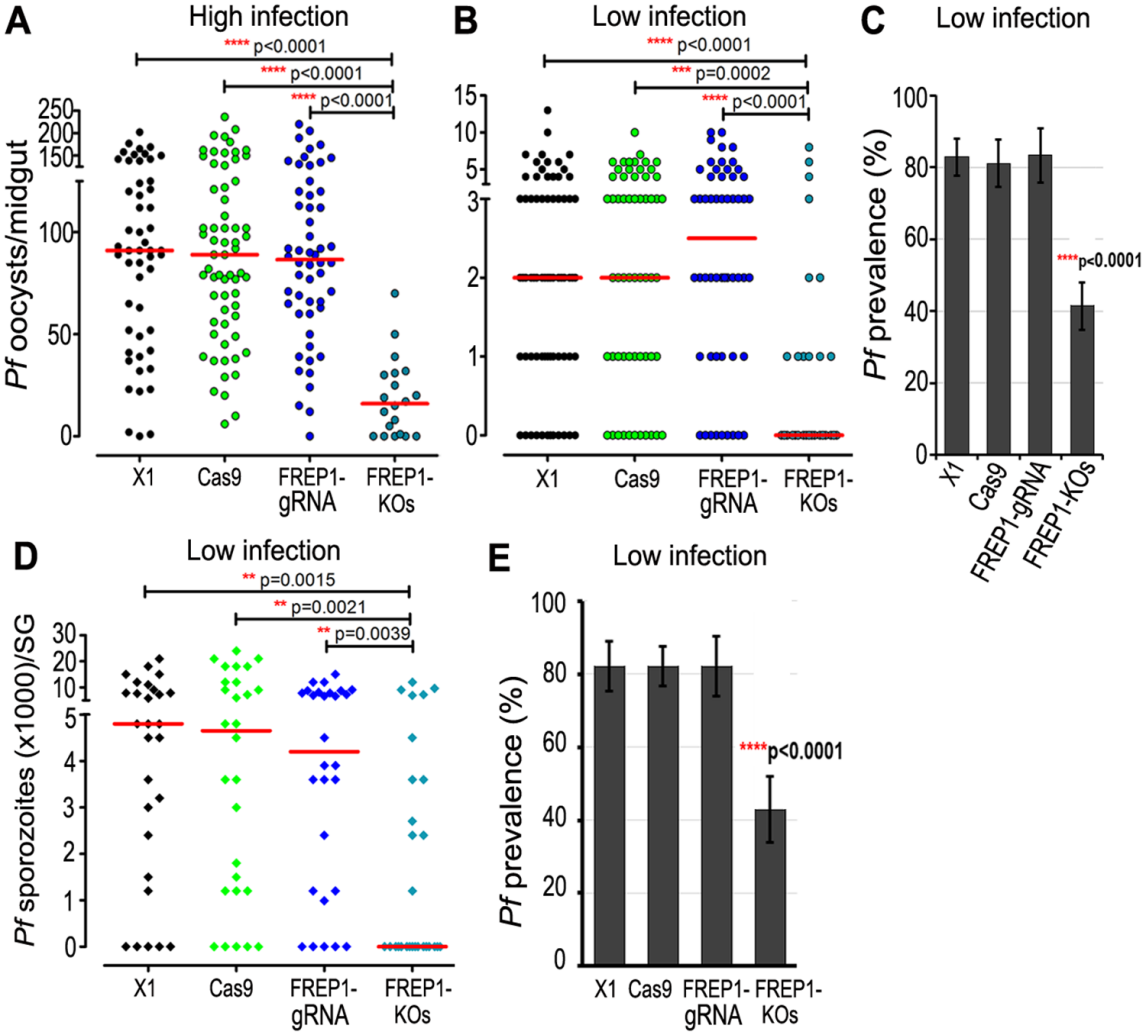
Suppression of *P*. *falciparum* in *FREP1* gene knockout mutants. (**A, B**) *P*. *falciparum* (NF54) oocyst infection intensities of *FREP1* knockout mutants at 8 days post-infection (dpi) when fed on blood with a high (0.1%) (**A**) or low (0.01%) (**B**) gametocytemia. *P*. *falciparum* oocyst infection intensity at 8 dpi in the wt (X1), *Vasa-Cas9* (Cas9), and FREP1-gRNA lines, and *FREP1* knockout mutants (FREP1-KOs). The wt (X1), Cas9, and FREP1-gRNA lines served as controls. (**C**) The prevalence of the oocyst infection was significantly lower in the *FREP1* knockout mosquitoes at low infection level. At low infection level, *P*. *falciparum* sporozoite loads in the salivary glands (SG) at 14 dpi (**D**) and the prevalence of the sporozoites (**E**) was significantly lower in the *FREP1* knockout mosquitoes. Assays were performed with at least three biological replicates, except for (**A**) (two replicates), and equal numbers of parasites from different replicates were pooled for the dot-plots. Each dot represents the number of parasites in an individual midgut or salivary gland, and the horizontal lines (red) indicate the median values. Mann-Whitney and Kruskal-Wallis (KW) tests were used to calculate *p*-values and determine the significance of parasite numbers. A chi-squared test was used to compare infection prevalence values. Detailed statistical analysis is presented in S2 Table.

To assess the effect of the *FREP1* gene knockout on *P*. *falciparum* infection, we fed the pool of homozygous *FREP1* mutants (to avoid any bias directed to certain single progenies), along with the X1, *Vasa-Cas9* (Cas9), and FREP1-gRNA strains as controls, on a NF54 *P*. *falciparum* gametocyte culture. Only fully engorged mosquitoes were included in the assay to ensure that the resulting differences in infection were derived from the *FREP1* gene knockout and not from potential changes in feeding behavior (discussed below). *FREP1* knockout mutants (FREP1-KOs) displayed significantly lower permissiveness to pre-oocyst stage *P*. *falciparum* when fed on blood with a high gametocytemia (0.1%), which resulted in an unnaturally high infection intensity (median of 91 oocysts per mosquito) and prevalence of 98.1% in wild-type (wt) mosquitoes (Fig 2A, S2 Table). The prevalence of infected mosquitoes (those with at least one oocyst on the midgut) decreased by 22.3% (chi-squared test, p<0.0001, S2 Table), and the median infection intensity was reduced by 81.3% when compared to wt X1 control mosquitoes (Kruskal-Wallis test, p<0.0001, Fig 2A, S2 Table). When the mosquito cohorts were fed on a 10-fold lower gametocytemia (0.01%), to mimic a natural infection system, the *FREP1* mutants displayed strong parasite suppression, with a median oocyst count of 0, as compared to the control mosquitoes that had a median oocyst count of 2.0 (X1 and Cas9 strains) and 2.5 (FREP1-gRNA strain). The *FREP1* knockout mutants (FREP1-KOs) displayed an infection prevalence of 41.3%, in contrast to 82.9% for the control X1 mosquitoes (Fig 2B and 2C and S2 Table). At low infection level, *FREP1* gene knockout also resulted in a profound decrease of the sporozoite-stage parasites in the salivary gland (Fig 2D), with a median sporozoite count of 0. The sporozoite infection prevalence showed a 1.9-fold decrease in the *FREP1* knockout mosquitoes (82.1% in the control mosquitoes vs 42.9% in the FREP1-KOs) (Fig 2E, S2 Table).

### Impact of *FREP*1 knockout on susceptibility to the rodent malaria parasite *P*. *berghei*

**Fig 3:**
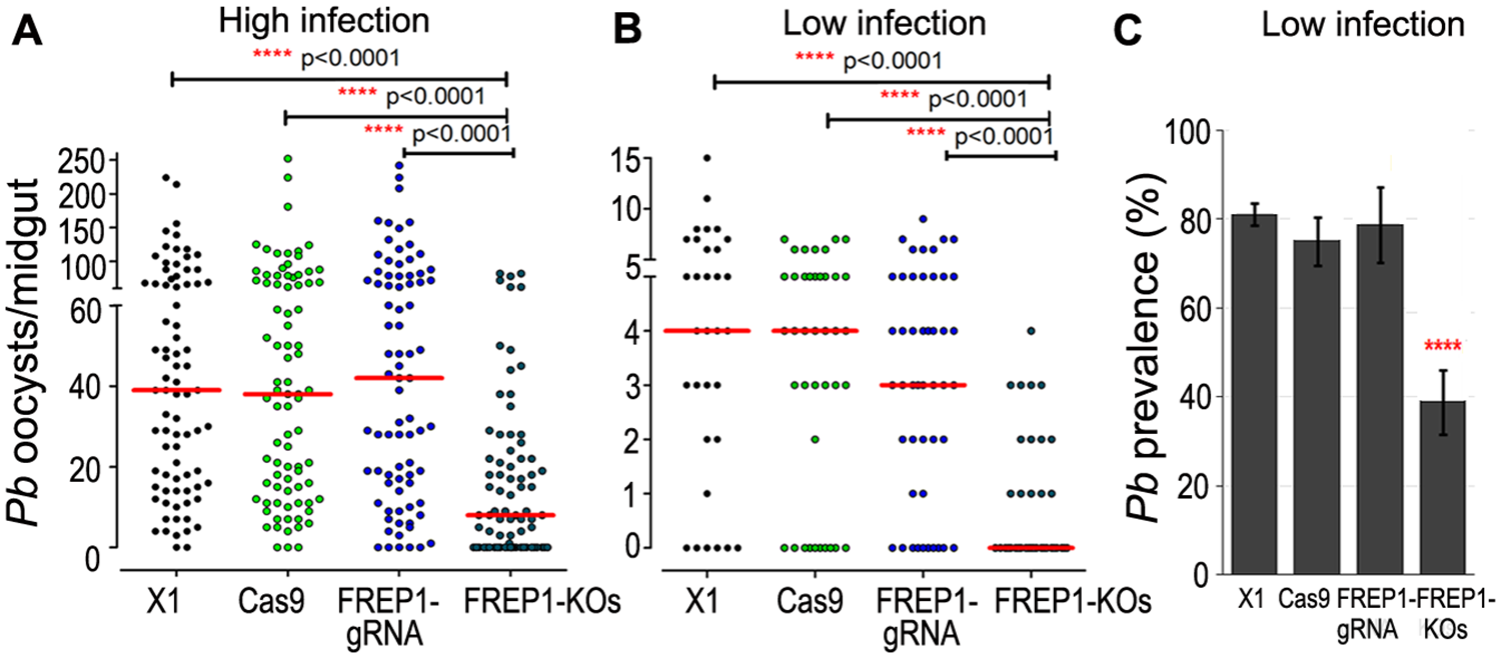
Suppression of *P*. *berghei* in CRISPR/Cas9-mediated *FREP1* gene knockout mutants. **(A, B)** *P*. *berghei* (wt, ANKA 2.34) oocyst infection intensities of FREP1 knockout mutants (FREP1-KOs) at 13 days post-infection when fed on *P*. *berghei*-infected mice at high (**A**) or low (**B**) infection level. wt (X1), Cas9, and FREP1-gRNA lines served as controls. (**C**) At low infection level the prevalence of the oocyst infection was significantly lower in the *FREP1* knockout mosquitoes. At least three biological replicates were included in each assay, and equal numbers of mosquitoes from the different replicates were pooled for the dot-plot analysis. Each dot represents the number of parasites in an individual mosquito, and the horizontal lines (red) indicate the medians. *p-*values were calculated using Mann-Whitney and Kruskal-Wallis (KW) tests. A chi-squared test was used to compare infection prevalence values. Detailed statistical analysis is presented in S3 Table.

To assess the permissiveness of *FREP1* knockout mosquitoes for the rodent malaria parasite *P*. *berghei*, we fed the knockout mosquitoes, along with the three control group mosquitoes (X1, Cas9, FREP1-gRNA), on *P*. *berghei* (wt, ANKA 2.34)-infected Swiss Webster mice. Only fully engorged mosquitoes were included in the assay to ensure that the differences observed in infection were related to the *FREP1* gene knockout and not to any change in feeding behavior (discussed below). Gene knockout of *FREP1* resulted in a significantly lower permissiveness to *P*. *berghei* oocyst infection: a 79.5% and 100% reduction in median infection intensity at high and low infection levels, respectively, when compared to the X1 control mosquitoes (Kruskal-Wallis test, p<0.0001, Fig 3A and 3B, S3 Table). At the high Infection level, the prevalence was 68.8% in the *FREP1* knockouts, vs. 97.5% for the control mosquitoes, while the infection prevalence showed a 2.1-fold decrease from 81.3% in the X1 control mosquitoes to 38.9% in the *FREP1* mutants at the low infection level (chi-squared test, p<0.0001, Fig 3C, S3 Table).

### Fitness impact of *FREP1* gene knockout

**Fig 4:**
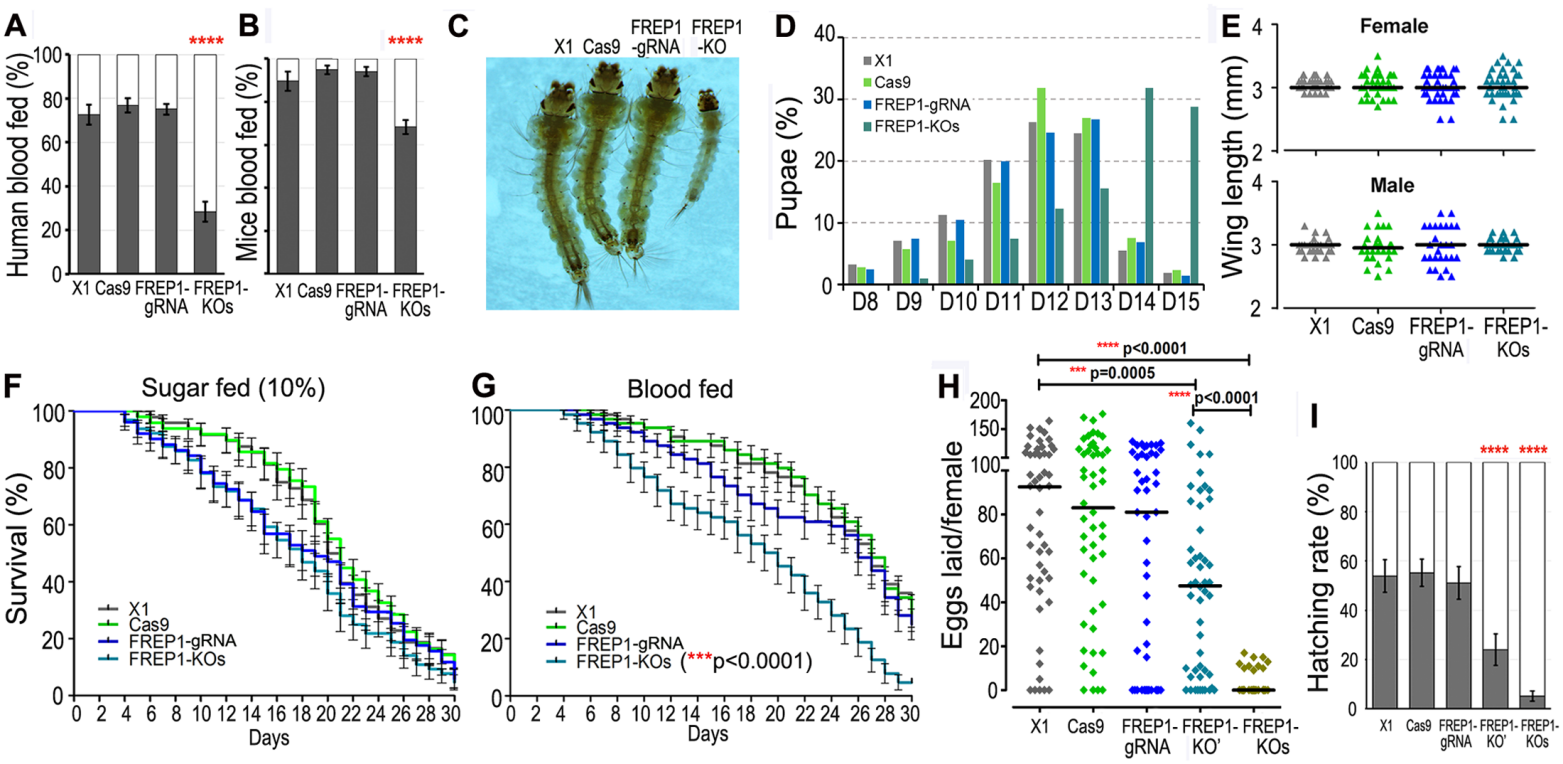
Fitness effects of *FREP1* gene knockout. **(A, B)** Percentage of mosquitoes fed on human blood through membrane (**A**) or naïve mice (**B**). A chi-squared test was used to compare blood feeding prevalence values. (**C**) *FREP1* gene knockout (KO) larva developed much more slowly than did control larvae. (**D**) The pupation time of the *FREP1* knockout mutants (*FREP1*-KOs) was significantly slower than that of the wt (X1), *Vasa-Cas9* (Cas9), or FREP1-gRNA control lines. The pupation time lagged behind the control time by a median of 2 days. (**E**) The wing lengths of the *FREP1* mutant females or males did not differ from those of the control mosquitoes. (**F, G**) Life spans of the *FREP1* mutants maintained on 10% sucrose solution (**F**) or after one mouse blood meal (**G**). The life spans of the mutants were significantly shorter than those of the controls when the mosquitoes were fed on the naïve mice. The pooled values from three replicates are shown, with standard error bars. Survival rates were analyzed by Kaplan-Meier survival analysis. (**H**) Numbers of eggs laid by female *FREP1* heterozygous mutants (FREP1-KO’) or homozygous mutants (FREP1-KOs) were significantly lower than those of the control transgenic lines. Each dot represents the eggs laid by an individual female after a single blood meal on mice. The median values (black horizontal bars) are shown. The *p*-values were calculated with a Mann-Whitney test. (**I**) Hatch rates indicate the average percentage of eggs giving rise to 1^st^ and 2^nd^ instar larvae, as determined by two replicates from two consecutive generations. Mean values for hatch rates and standard errors (SE) of replicates are indicated. ****, p<0.0001.

To determine whether *FREP1* knockout influences the blood-feeding ability of mosquitoes, we first exposed the *FREP1* knockout mutants and the control (X1, Cas9, FREP1-gRNA) strains to blood-containing membrane feeders, or anesthetized mice, for at least 45 min. All the mosquitoes had been starved of sugar for 3~5 h prior to the assays. *FREP1* knockout mosquitoes showed a 2.6-fold lower feeding propensity when compared to the controls; only 28% of the *FREP1* knockout mosquitoes fed on the human blood-containing membrane feeders, in contrast to 73% of the wt (X1) control mosquitoes (Fig 4A). Similarly, *FREP1* knockout mosquitoes showed a 1.3-fold lower feeding propensity when exposed to mice, with 68% of the *FREP1* knockout mosquitoes feeding on the mice, as compared to 90%~95% of the X1 control mosquitoes (Fig 4B).

Next we compared the larval development and pupation time of the *FREP1* knockout mutants to that of the control strain mosquitoes and observed a retarded larval development of the *FREP1* knockout mutants. When 90% of the control mosquito larvae had reached the L4 stage, all of the knockout mutants were still at L2 stage (Fig 4C). The average pupation time of the knockout mutants was 2.0 days longer than that of the control strains (Kruskal-Wallis test, p<0.0001) (Fig 4D). However, the adult *FREP1* knockout mosquitoes that emerged showed no difference in their body size from those of the control strains, as measured by the wing lengths in both female and male mosquitoes (Fig 4E). The life span of the adult *FREP1* knockout mosquitoes did not differ from that of the control strains under laboratory rearing conditions, when mosquitoes were maintained on a 10% sugar solution in the absence of a blood meal (Fig 4F). Exposure of mosquitoes to a single naïve blood meal resulted in an increased longevity of the control mosquitoes but not of the *FREP1* knockout mosquitoes (Kaplan-Meier survival analysis, p<0.0001, Fig 4G).

Fecundity, as a measure of egg-laying capacity, was significantly lower for the heterozygous and homozygous *FREP1* knockout mosquitoes than for the controls (Mann-Whitney test, p<0.0001, Fig 4H). The egg hatch rate was also significantly lower for the heterozygous and homozygous *FREP1* mutants (chi-squared test, p<0.0001, Fig 4I).

## Discussion

Here we have, for the first time, created an *A*. *gambiae Plasmodium* host factor gene knockout mosquito using CRISPR/Cas9 genome editing. We targeted the mosquito-encoded *Plasmodium* host factor FREP1, which plays an essential role during the parasite’s midgut infection stage. FREP1 had previously been investigated for its role as a *Plasmodium* host factor by using RNAi-mediated gene silencing methodology and FREP1-inhibiting polyclonal antibodies, and these studies showed that it represents a host factor for *P*. *falciparum*, *P*. *vivax*, and *P*. *berghei*. However, *FREP1* silencing resulted in an average 50% and 11% decrease in *P*. *falciparum* infection intensity and prevalence, respectively, at a high infection level (with median oocyst counts of 50 and 20 in two replicates) [21]. The complete inactivation of the *FREP1* gene using gene editing showed a much stronger suppression of *Plasmodium*, most likely because RNAi-based gene silencing results in only partial protein depletion. The refractoriness level of *FREP1* knockout mosquitoes to *P*. *falciparum* was similar to that achieved when the anti-*Plasmodium* IMD pathway transcription factor *Rel2* was transgenically over-expressed after a blood meal, or when the insulin pathway was transgenically activated [25, 29]. Parasite suppression at the sporozoite stage in the *FREP1* knockout mosquitoes suggests that an epidemiologically significant impact would be achieved if these mosquitoes would replace wt mosquitoes and were able to persist in a malaria-endemic region [30–32].

Significant advances in the development of *A*. *gambiae* gene drives has been achieved, aimed at the development of mosquito population replacement methods in which a mosquito mutation, or transgene, causing resistance to the parasite could be propelled and spread into a population of susceptible mosquitoes. However, depending on the efficiency of a gene-drive mechanism, a strong fitness impact of the mutations and transgenes could make this approach unfeasible for spreading transgenic malaria-resistance into a mosquito population [33]. Mosquito host factors for *Plasmodium* often play essential biological roles, and their complete inactivation at all developmental stages and in all tissues would therefore be expected to adversely affect fitness. Consistent with this expectation, the *FREP1* knockout mosquitoes showed a diminished feeding propensity, a slower pre-adult development, and decreased fecundity; furthermore, a blood meal failed to improve their longevity. All these fitness effects may be related to FREP1’s role in blood feeding and digestion as a putative peritrophic matrix factor [21] since its inactivation could result in decreased nutritional uptake or in impaired midgut protection against stress. The peritrophic matrix is a key regulator of mosquito gut homeostasis [34] and nutritionally deprived female mosquitoes may lay fewer eggs with decreased hatching rate and prolonged larval developing time. Several fibrinogen (FBG) domain-containing proteins have been shown to play roles in processes taking place in the blood. For example, the vertebrate fibrinogen (FBG) proteins, ficolins, are involved in blood clotting [35–36]. The function of FREPs in the snail hemolymph is analogous to that of calcium-dependent lectins [37–38]. The snake C-type lectin binds specifically with activated factor X, which functions as anticoagulation factor [39]. FREP1 may also play diverse roles in mosquito biology having similar functions as other FBG-containing proteins in other organisms, and this gene’s inactivation could thereby have directly impacted on various developmental processes. In addition, CRISPR/Cas9 mediated gene editing is known for creating unwanted changes at non-target sites though we have selected the gRNA targets with the least potential off-target sites, and such off-target effects might also have contributed to the fitness impairment [40–42], although most off-target mutations would not be expected to become homozygous mutants during the process of the enrichment of a homozygous mosquito population through PCR and sequencing unless they were genetically tightly linked to *FREP1*. These types of fitness effects are likely to occur after germline-targeted deletion of the majority of *Plasmodium* host factors, but they could to some extent be overcome by bloodmeal-inducible adult stage- and tissue-specific gene deletion systems. This challenge could be met through the conditional knockout of *Plasmodium* host factors via blood-meal inducible expression of either the Cas9 protein or gRNAs in the midgut or salivary gland tissues [43]. It is noteworthy that studies with transgenic *Anopheles* that over-express certain anti-*Plasmodium* immune factors in a tissue- and bloodmeal-inducible manner do not show measurable fitness effects under laboratory conditions; rather, they show a fitness advantage related to the rapid spread of the transgene in a cage population [25, 44]. However, given the likelihood of mosaic expression of Cas9 protein and gRNA target sequences in different cell types of the same tissue, and the diverse and numerous mutations occurring upon somatic mutation, the effectiveness of conditional CRISPR/Cas9 gene knockout of host factors in blocking the transmission of *Plasmodium* parasites warrants further studies. Nevertheless, the deletion of *Plasmodium* host factors remains a potentially powerful approach for the study of their biological function and ability to influence infection, as well as for the generation of *Plasmodium*-resistant mosquitoes that can compete with wild mosquitoes, and further studies are required to explore this approach.

## Methods

### Ethics statement

This study was carried out in strict accordance with the recommendations in the Guide for the Care and Use of Laboratory Animals of the National Institutes of Health. The protocol was approved by the Animal Care and Use Committee of the Johns Hopkins University (permit number MO15H144). Commercial anonymous human blood was used for parasite cultures and mosquito feeding, and informed consent was therefore not applicable. The Johns Hopkins School of Public Health Ethics Committee has approved this protocol.

### gRNA target design

For the design of guide RNA (gRNA) targets, manually searching the *FREP1* coding sequence for the PAMs (NGG) was initially undertaken, with N representing any nucleotide. We checked candidate gRNA sequences for potential off-target binding using the following web tools: http://zifit.partners.org/ZiFiT/, http://crispr.mit.edu/, http://www.rgenome.net/cas-designer/, and http://chopchop.cbu.uib.no/, and retained three 20-nucleotide specific target sequences that were followed by a PAM and had the fewest predicted genomic off-targets. The final selected gRNA target sequences are within the RNAi primers region and start with a G considering the transcription from U6 promoter starts with a G (S1 Table).

### Generation of U6-gRNAs constructs via the Golden Gate assembly system

We selected three gRNA targets for the *FREP1* gene, and the forward and reverse primers for each gRNA target DNA sequence (AgFREP1-gRNA1 –F and –R for gRNA1, AgFREP1-gRNA2 –F and –R for gRNA2, and AgFREP1-gRNA3 –F and –R for gRNA3 target) were synthesized by IDT DNA (S1 Table). The two primers of each pair are complementary to each other over the 20 nt specific to the chosen gRNA target and their annealing produces a linker with specific overhangs for the two *Bbs*I sites of the cloning vectors. Three pKSB-sgRNA vectors (pKSB-sgRNA-1, -2, -3) with U6 snRNA polymerase III promoter (AGAP013557), CRISPR RNA invariable sequences, and the RNA polIII TTTTT terminator (U6-Term, Figs 1A and S1) sequences were constructed in a pBluescript cloning vector backbone. U6::gRNA constructs were generated by cloning gRNA target DNA sequences -1, -2, and -3 separately into the *Bbs*I site on these three pKSB-sgRNA vectors followed by sequencing confirmation. At least two clones from each construct were selected for mini-prep analysis and sequencing to identify the positive clones. The sequencing confirmed three pKSB-FREP1-gRNA plasmids with three gRNA target DNA sequences against *FREP1* were assembled into the destination pDSAT vector through Golden Gate cloning into the *Bsa*I sites, for embryo microinjection of the *A*. *gambiae* docking line X1 (Fig 1A) [28]. The final plasmid pDSAT-FREP1-gRNA_3_, with 3xP3-TFP (mTurquoise2) eye-specific fluorescence markers, allowed us to screen the positive G_0_ (for the transient expression of transgene) and G_1_ larvae or mosquitoes by using blue fluorescence (Fig 1B and 1C).

### *A*. *gambiae* embryo microinjection and screening for gRNA-expressing transgenic line

The construct (pDSAT-FREP1-gRNA_3_) described above and a helper plasmid carrying the Vasa2::ΦC31-Integrase gene (pENTR-R4R3-Vasa2-integrase) [24, 28] were prepared with a Qiagen Endofree Maxi Kit before embryo microinjection according to the established protocols [25, 27–28] (S1 Fig). Freshly laid eggs of docking line X1 were directly aligned against the edge of a nylon membrane that was kept wet with overlaid filter paper soaked with distilled (DI) water, and a mix of the transgenesis plasmid (160 ng/μL) and the integrase helper plasmid (200 ng/μL) in phosphate buffer (0.1 mM NaHPO4 buffer, 5 mM KCl, pH 6.8) was injected into approximately 800 embryos of docking strain X1 [28, 45]. These injected eggs were removed from the slides and kept on a piece of DI water-wetted slanted filter paper (one end of the paper was submerged in the DI water to retain moisture) for 2 days in the insectary before hatching. The hatched G_0_ larvae were screened for transient fluorescence expression at the second instar larval (L2) stage by observation with a fluorescence stereomicroscope. Approximately 28% of the hatched larvae showed transient expression of the reporter gene, and the surviving transiently positive G_0_ pupae were sexed first (16 females and 12 males) and then organized into two cohorts. G_0_ female mosquitoes were crossed with wt (X1) male mosquitoes at a ratio of 1:1, and the surviving G_0_ male mosquitoes were crossed with female X1 mosquitoes at a ratio of 1:5. The G_1_ progeny were examined for blue fluorescent glowing eyes at both the larval and adult stages (Fig 1B and 1C). *FREP1-gRNA*-expressing transgenic positive progeny was outcrossed with X1 for two generations followed by two generations of self-crossing to enrich the homozygous mosquitoes by screening the fluorescence in the larvae before crossing with the *Vasa-Cas9* strain to generate germ-line knockout mutants (S1 Fig).

### Generation of CRISPR/Cas9 knockout mutants and molecular confirmation of CRISPR activity at the target loci

The G_4_ (fourth-generation) FREP1-gRNA lines were crossed with mosquitoes expressing the Cas9 protein in the germ-line (*Vasa2-Cas9*; with yellow (YFPvenus) fluorescence in the eyes) [16] (S1 Fig). We crossed about 100 virgin FREP1-gRNA female mosquitoes with 50 male *Vasa-Cas9* mosquitoes to generate knockout mutants. Since the parental gRNA mosquitoes were not fully homozygous, we eliminated all the larvae that did not carry the FREP1-gRNA sequences by retaining only larvae with both transgenes (*FREP1-gRNA/Cas9* with both blue and YFP fluorescence [Fig 1D]). The males from this transheterozygous *FREP1-gRNA/Cas9* (FREP1/Cas9) culture were then crossed with wt virgin females. Among the progeny of this new cross, only the virgin females (potential heterozygous mutants) were selected and crossed with wt males. This final cross was then fed on naïve mice, and 3 days post-blood feeding, each individual female mosquito was placed in a single tube (50 ml Falcon tube with 1 cm of water and a conical Whatman filter surrounding the plastic walls) for these female mosquitoes to lay eggs. Two days later, each female that produced eggs was collected in a single Eppendorf tube with 100 μl of lysis buffer for genomic DNA (gDNA) extraction. The mutant region from the mother was amplified by PCR with flanking primers (S1 Table). PCR products were run on a 1.2% agarose gel to visualize both the mutant band (with a deletion between FREP1-gRNA1 and FREP1-gRNA2 targets) and the wt band, since the mothers were heterozygous. PCR products were also sequenced to confirm the deletion (SI S1 File). The progenies from each mother were kept as separate families. Among the 50 founder mothers, 5 showed the same 155 bp deletion mutation, and 12 (sub-grouped into 4 different sequence variations) showed smaller deletions which were confirmed by sequencing. After sequencing confirmation of the mutants, the separate progenies from mutant mothers were selected and sexed at the pupal stage to separate males and females before mating. PCR on one leg (Phire kit with dilution protocol) was used to identify the mutant mosquitoes, and all the mutant mosquitoes with the same genotype were pooled. This process was repeated in the next generation to enrich for homozygous mutants. gDNA was prepared as previously described [25, 27]. PCR with FREP1-KO-seqF1 and FREP1-KO-seqR3 primers (S1 Table, S1 Fig) was used to amplify a 478 bp fragment for sequencing. The mutation of the gene was determined by alignment of the mutant sequence with the wt sequence through Blast2 (S1 File).

### *P*. *falciparum* and *P*. *berghei* infection assays

To determine the anti-*Plasmodium* activity, the *FREP1* knockout mutants and wt X1, *Vasa-Cas9* (Cas9), and FREP1-gRNA lines were fed on NF54 *P*. *falciparum* gametocyte cultures (provided by the Johns Hopkins Malaria Institute Core Facility; NF54 obtained from MR4) through artificial membranes at 37°C or on a *P*. *berghei* wt ANKA 2.34-infected Swiss Webster mouse (at 19°C) [18, 25]. The adult mosquitoes were starved for 3 to 5 h prior to feeding to ensure engorgement. Unfed mosquitoes were removed immediately after blood feeding, and the remaining were incubated for a further 8 days at 27°C or 13 days at 19°C for *P*. *falciparum* and *P*. *berghei*, respectively. Midguts were dissected in PBS, stained with 0.2% mercurochrome, and examined using a phase-contrast microscope (Leica). At least two to three biological replicates were performed for each experiment. As described previously, after comparing the variance in oocyst numbers between different replicates, equal numbers of midguts from the various replicates were selected randomly and pooled for producing dot-plots using GraphPad Prism 7 software [25, 46–47]. A published method [25, 48] was used, with some modifications, to determine the sporozoite loads in the salivary glands of the infected mosquitoes. Salivary glands were dissected, and individual glands were placed in Eppendorf tubes with 30 μl of PBS, followed by homogenization on ice. A sample (10 μl) of this homogenate was placed in a Neubauer counting chamber and counted after 5 min using a Leica phase-contrast microscope at 400x magnification.

The dot plots of oocyst and sporozoite numbers in each individual gut and salivary gland were generated using GraphPad Prism 7 software, along with the median values, represented as red bars. The p-values were calculated using a non-parametric Mann-Whitney test or Kruskal-Wallis ANOVA on ranks and used to determine the significance of differential infection levels as described in [18, 25, 46]. Chi-squared analysis was used to determine the significance of infection prevalence [29, 46]. To gain a more detailed picture of prevalence and infection intensity at both high and low infection intensity levels, we have separated these two attributes into two data sets for the statistical analysis, and calculated p-values with or without uninfected mosquitoes as shown in S2 and S3 Tables. Median, range of infection, and N numbers are presented in S2 and S3 Tables.

### Wing length, pupation time, life span, fecundity, and egg hatching rate measurements

Adult wing length (both male and female) was used as a surrogate measurement for mosquito size. Mosquitoes were anesthetized on ice and kept on a cold plate for wing length measurement. Wing length was measured manually from the distal end of the alula to the tip of the wing (without the hairy fringe) through a microscope objective containing a scale bar calibrated to a 1-mm stage micrometer without taking pictures or using software [44].

Larvae were maintained according to a standard procedure with 400 larvae in each tray, and the number of pupae present each day was recorded on a daily basis to determine development time [44]. At least three biological replicates were included for each strain.

In order to measure the life span of the various mosquito lines, adult mosquitoes were placed into cups within 12 h of emergence with a cotton pad constantly impregnated with a 10% sucrose solution. They were held there until all the mosquitoes in that cup died, and the number of dead mosquitoes in the cup was recorded and the dead mosquitoes removed daily [25, 44]. For the life span assays of mosquitoes receiving a single blood meal (blood-fed on mice), the mosquitoes were offered a blood meal 5 days after emergence, and only mosquitoes taking a blood meal on day 5 were kept for the rest of the study. The survival percentage represents the mean survival percentage for all three biological replicates of 35 mosquitoes each. Statistical significance was determined by Kaplan-Meier survival analysis with pooled data from three replicates by using GraphPad Prism 7 software, and *p*-values were determined by Wilcoxon test as described in [25, 29].

For the fecundity assay, approximately 50 7-day-old adult female wt X1, *Vasa-Cas9*, FREP1-gRNA, and heterozygous or homozygous *FREP1* knockout mutant mosquitoes were allowed to feed on mice. Mosquitoes were anesthetized on ice immediately following the blood meal, and all non-engorged mosquitoes were discarded. At 2 days post-blood feeding, female mosquitoes were separated into individual vials (50 ml Falcon tubes) containing moist filter paper with 1 ml of water on the bottom and allowed to oviposit, and the number of eggs laid by each female was recorded 4 days after the blood feeding using light microscopy. The females that died before laying eggs were excluded from the assays. After each count, the eggs were submerged in larval rearing water in the same individual tubes to allow eggs to hatch. First- and second- instar larvae were counted under a light microscope. The fecundity and larval hatch-rate assays were performed for two consecutive generations (two biological replicates), and the number of eggs laid by each female and their hatch rates were pooled to calculate the median value. Statistical significance was determined using the Mann-Whitney test with GraphPad Prism 7 software. The control mosquitoes were reared under the same conditions as the mutants.

## Supporting information

S1 Fig Generation of *FREP1-gRNA-*expressing transgenic line and homozygous *FREP1* knockout mutants. The generation of *FREP1*-*gRNA-*expressing transgenic line and homozygous *FREP1* knockout mutants is outlined.
(TIF)

S1 Table List of gRNA target sequences and primers used in the study. Primers used for generation of constructs for embryo microinjections and the verification of transgene integration and gene knockout.
(DOCX)

S2 Table *P*. *falciparum* infection data. Additional statistical analysis of numbers of *P*. *falciparum* parasites for Fig 2 (Mann-Whitney, Kruskal-Wallis test, chi-squared test).
(DOCX)

S3 Table *P*. *berghei* infection data. Additional statistical analysis of numbers of *P*. *berghei* parasites for Fig 3. (Mann-Whitney, Kruskal-Wallis test, chi-squared test).
(DOCX)

S1 File Sequences. Sequences related to Fig 1F and *FREP1* Knockouts.
(DOCX)
