# Supplementary figures and images for: CRISPR/Cas9 -mediated gene knockout of Anopheles gambiae FREP1 suppresses malaria parasite infection
Source: PLoS Pathog. 2018 Mar 8;14(3):e1006898. doi: 10.1371/journal.ppat.1006898 (PMC5843335; doi:10.1371/journal.ppat.1006898)

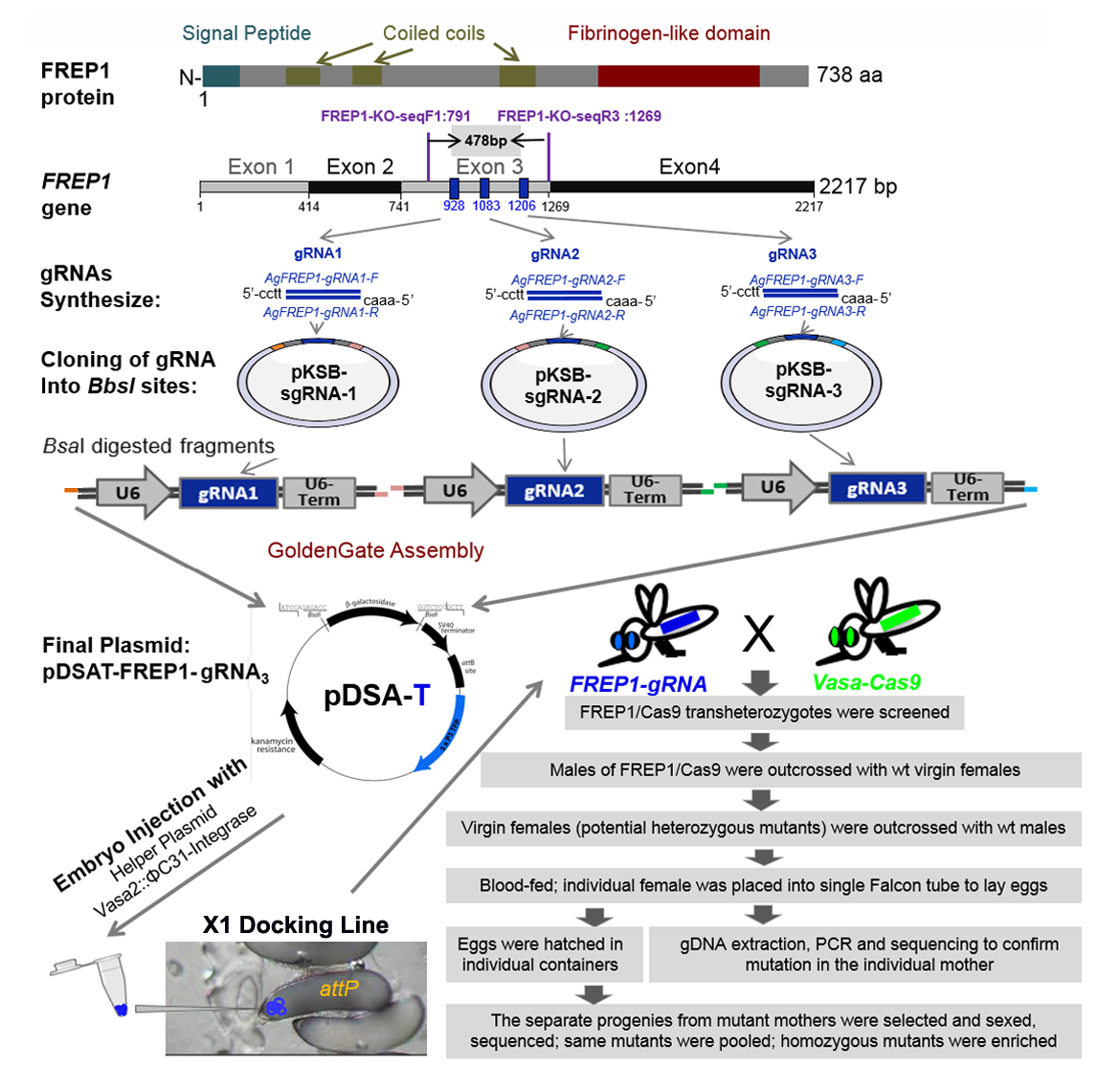

Supplement: S1 Fig — The generation of FREP1-gRNA-expressing transgenic line and homozygous FREP1 knockout mutants is outlined. (TIF) [file ppat.1006898.s001.tif]
