# Supplementary material for: CRISPR/Cas9 -mediated gene knockout of Anopheles gambiae FREP1 suppresses malaria parasite infection
Source: PLoS Pathog. 2018 Mar 8;14(3):e1006898. doi: 10.1371/journal.ppat.1006898 (PMC5843335; doi:10.1371/journal.ppat.1006898)
Supplement: S1 Table — Primers used for generation of constructs for embryo microinjections and the verification of transgene integration and gene knockout. (DOCX) [file ppat.1006898.s002.docx]

**Table S1. List of gRNA target sequences and primers used in the study.** Primers used for generation of constructs for embryo microinjections and the verification of transgene integration and gene knockout.

| **Name** | **Sequence (with PAM)** | **Cleavage Position (%)*** | **Direction*** | **GC Contents (%, w/o PAM)*** | **Out-of-frame Score*** | **Mismatches*** | | | **Genomic location^#^** | **Exon^#^** | **Self-complementarity^#^** | **Off-targets^#^** | | | | **Efficiency^#^** |
| --- | --- | --- | --- | --- | --- | --- | --- | --- | --- | --- | --- | --- | --- | --- | --- | --- |
|  |  |  |  |  |  | **0** | **1** | **2** |  |  |  | **0** | **1** | **2** | **3** |  |
| gRNA1 | GCCGGGCTGGACAAGCTGCACGG | 51.6 | + | 70 | [50.1](javascript:%20void(0);) | [1](javascript:%20void(0);) | 0 | 0 | 2L:41177106 | 3 | 1 | 0 | 0 | 0 | 3 | 0.5 |
| gRNA2 | GCGGGAGGAGGTGCACAACGCGG | 74.8 | + | 70 | [58.6](javascript:%20void(0);) | [1](javascript:%20void(0);) | 0 | 0 | 2L:41176951 | 3 | 0 | 0 | 0 | 0 | 1 | 0.73 |
| gRNA3 | GCTGGCCCGGATCCGGTCCGCGG | 93.3 | + | 80 | [64.9](javascript:%20void(0);) | [1](javascript:%20void(0);) | 0 | 0 | 2L:41176828 | 3 | 2 | 0 | 0 | 0 | 0 | 0.62 |

| **Primer Name** | **Purpose** | **Sequence** |
| --- | --- | --- |
| AgFREP1-gRNA1-F | cloning of gRNA1 target DNA sequences to pKSB-sgRNA-1 | ccttGCCGGGCTGGACAAGCTGCA |
| AgFREP1-gRNA1-R | cloning of gRNA1 target DNA sequences to pKSB-sgRNA-1 | aaacTGCAGCTTGTCCAGCCCGGC |
| AgFREP1-gRNA2-F | cloning of gRNA2 target DNA sequences to pKSB-sgRNA-2 | ccttGCGGGAGGAGGTGCACAACG |
| AgFREP1-gRNA2-R | cloning of gRNA2 target DNA sequences to pKSB-sgRNA-2 | aaacCGTTGTGCACCTCCTCCCGC |
| AgFREP1-gRNA3-F | cloning of gRNA3 target DNA sequences to pKSB-sgRNA-3 | ccttGCTGGCCCGGATCCGGTCCG |
| AgFREP1-gRNA3-R | cloning of gRNA3 target DNA sequences to pKSB-sgRNA-3 | aaacCGGACCGGATCCGGGCCAGC |
| FREP1-KO-seqF1 | PCR primers for confirmation of mutation sequences | AGCTCGAGGTGAAGCAGAGCG |
| FREP1-KO-seqF2 | PCR primers for confirmation of mutation sequences | CGTCGAGATCGAGCAGCGCCG |
| FREP1-KO-seqF3 | PCR primers for confirmation of mutation sequences | CAACGTCAGCCGACAGATCGC |
| FREP1-KO-seqR1 | PCR primers for confirmation of mutation sequences | CGCTGACCGCCATCGCCTGGA |
| FREP1-KO-seqR2 | PCR primers for confirmation of mutation sequences | ATCTCGCGGTGCAGCACGGCC |
| FREP1-KO-seqR3 | PCR primers for confirmation of mutation sequences | TCTCCAGCCGGTGTGTGCCAT |

*: from <http://www.rgenome.net/cas-designer/>

^#^: from <http://chopchop.cbu.uib.no/>
