# Supplementary material for: CRISPR/Cas9 -mediated gene knockout of Anopheles gambiae FREP1 suppresses malaria parasite infection
Source: PLoS Pathog. 2018 Mar 8;14(3):e1006898. doi: 10.1371/journal.ppat.1006898 (PMC5843335; doi:10.1371/journal.ppat.1006898)
Supplement: S2 Table — Additional statistical analysis of numbers of P. falciparum parasites for Fig 2 (Mann-Whitney, Kruskal-Wallis test, chi-squared test). (DOCX) [file ppat.1006898.s003.docx]

**Table S2. *P. falciparum* infection data.** Additional statistical analysis of numbers of *P. falciparum* parasites for **Fig 2** (Mann-Whitney, Kruskal-Wallis test, chi-squared test).

| ***Fig. 2A (Pf oocysts) High*** | **X1** | **Cas9** | **FREP1-gRNA** | **FREP1-KOs** |
| --- | --- | --- | --- | --- |
| n= | 52 | 64 | 54 | 20 |
| range | 0-202 | 0-236 | 0-220 | 0-70 |
| prevalence | 98.1% | 100.0% | 98.2% | 76.2% |
| Chi-square test p-value |  |  |  | <0.0001 (****) |
| median (with zeros) | 91.0 | 89.0 | 88.0 | 17.0 |
| % decreased median oocysts# |  |  |  | 81.3% |
| Kruskal-Wallis p-value |  |  |  | <0.0001 (****) |
| Mann-Whitney test p-value | < 0.0001 | < 0.0001 | < 0.0001 |  |
| median (without zeros) | 91.0 | 89.0 | 86.5 | 20.0 |
| Kruskal-Wallis p-value |  |  |  | <0.0001 (****) |
| Mann-Whitney test p-value | < 0.0001 | < 0.0001 | < 0.0001 |  |
| ***Fig. 2B&C (Pf oocysts) Low*** | **X1** | **Cas9** | **FREP1-gRNA** | **FREP1-KOs** |
| n= | 72 | 57 | 54 | 30 |
| range | 0-13 | 0-10 | 0-10 | 0-8 |
| prevalence | 82.9% | 81.0% | 83.3% | 41.3% |
| Chi-square test p-value |  |  |  | <0.0001 (****) |
| median (with zeros) | 2.0 | 2.0 | 2.5 | 0.0 |
| % decreased median oocysts# |  |  |  | 100.0% |
| Kruskal-Wallis p-value |  |  |  | <0.0001 (****) |
| Mann-Whitney test p-value | < 0.0001 | 0.0002 | < 0.0001 |  |
| median (without zeros) | 2.0 | 3.0 | 3.0 | 1.5 |
| Kruskal-Wallis p-value |  |  |  | 0.1981 |
| Mann-Whitney test p-value | 0.11375 | 0.0684 | 0.03125 |  |
| ***Fig. 2D&E (Pf sporozoites)*** | **X1** | **Cas9** | **FREP1-gRNA** | **FREP1-KOs** |
| n= | 28 | 28 | 28 | 28 |
| range | 0-21000 | 0-24000 | 0-15000 | 0-12000 |
| prevalence | 82.1% | 82.1% | 82.1% | 42.9% |
| Chi-square p-value |  |  |  | <0.0001 (****) |
| median (with zeros) | 4800 | 4650 | 4200 | 0 |
| % inhibition of median sporozoites |  |  |  | 100.0% |
| Kruskal-Wallis p-value |  |  |  | 0.003 |
| Mann-Whitney test p-value | 0.0015 | 0.0021 | 0.0039 |  |
| median (without zeros) | 5800 | 6000 | 6600 | 4050 |
| Kruskal-Wallis p-value |  |  |  | 0.6165 |
| Mann-Whitney test p-value | 0.1149 | 0.1562 | 0.271 |  |
