# Supplementary material for: CRISPR/Cas9 -mediated gene knockout of Anopheles gambiae FREP1 suppresses malaria parasite infection
Source: PLoS Pathog. 2018 Mar 8;14(3):e1006898. doi: 10.1371/journal.ppat.1006898 (PMC5843335; doi:10.1371/journal.ppat.1006898)
Supplement: S3 Table — Additional statistical analysis of numbers of P. berghei parasites for Fig 3. (Mann-Whitney, Kruskal-Wallis test, chi-squared test). (DOCX) [file ppat.1006898.s004.docx]

**Table S3. *P. berghei* infection data.** Additional statistical analysis of numbers of *P. berghei* parasites for **Fig 3.** (Mann-Whitney, Kruskal-Wallis test, chi-squared test).

| ***Fig. 3A (Pb oocysts) High*** | **X1** | **Cas9** | **FREP1-gRNA** | **FREP1-KOs** |
| --- | --- | --- | --- | --- |
| n= | 81 | 84 | 81 | 78 |
| range | 0-224 | 0-252 | 0-242 | 0-82 |
| prevalence | 97.5% | 96.4% | 93.8% | 68.8% |
| Chi-square test p-value |  |  |  | 0.0001 (****) |
| median (with zeros) | 39.0 | 38.0 | 42.0 | 8.0 |
| % decreased median oocysts# |  |  |  | 79.5% |
| Kruskal-Wallis p-value |  |  |  | <0.0001 (****) |
| Mann-Whitney test p-value | < 0.0001 | < 0.0001 | < 0.0001 |  |
| median (without zeros) | 39.0 | 39.0 | 45.0 | 18.0 |
| Kruskal-Wallis p-value |  |  |  | <0.0001 (****) |
| Mann-Whitney test p-value | < 0.0001 | < 0.0001 | < 0.0001 |  |
| ***Fig. 3B&C (Pb oocysts) Low*** | **X1** | **Cas9** | **FREP1-gRNA** | **FREP1-KOs** |
| n= | 33 | 39 | 48 | 36 |
| range | 0-15 | 0-7 | 0-9 | 0-4 |
| prevalence | 81.3% | 75.0% | 78.7% | 38.9% |
| Chi-square p-value |  |  |  | <0.0001 (****) |
| median (with zeros) | 4.0 | 4.0 | 3.0 | 0.0 |
| % decreased median oocysts# |  |  |  | 100.0% |
| Kruskal-Wallis p-value |  |  |  | <0.0001 (****) |
| Mann-Whitney test p-value | < 0.0001 | < 0.0001 | < 0.0001 |  |
| median (without zeros) | 5.0 | 5.0 | 4.0 | 2.0 |
| Kruskal-Wallis p-value |  |  |  | <0.0001 (****) |
| Mann-Whitney test p-value | < 0.0001 | < 0.0001 | < 0.0001 |  |
