# Supplementary material for: CRISPR/Cas9 -mediated gene knockout of Anopheles gambiae FREP1 suppresses malaria parasite infection
Source: PLoS Pathog. 2018 Mar 8;14(3):e1006898. doi: 10.1371/journal.ppat.1006898 (PMC5843335; doi:10.1371/journal.ppat.1006898)
Supplement: S1 File — Sequences related to Fig 1F and FREP1 Knockouts. (DOCX) [file ppat.1006898.s005.docx]

**S1 File. Sequences.** Sequences related to Fig 1F and *FREP1* Knockouts.

**>X1-FREP1’** (478bp PCR fragment from X1 wt mosquito)

AGCGAACTGCACGGCCACCGGCTAGCGGACCGGCTGCGCAGCGTCGAGATCGAGCAGCGCCGCCTTGCGGGCGCCAACTTCAACGTCAGCCGACAGATCGCCGGGCTGGACAAGCTGCACGGCTCGATGCTGGAGCTGCTCGAGGACGTGGAAGCGATACAGGGCAAGTTCGAGAAGACGGTACCGGACATGCGGCGCGAAATCGCCAAGGTAGAGTTCAGCGTCGCGCAGGCCGCCTCCGAGCAGGGGCTGGTGCGGGAGGAGGTGCACAACGCGGCCAAAAGCATCCAGGCGATGGCGGTCAGCGTGAGCGCGCTGCAGGAGGAGCGCGACACGGTCAAGCGGCTGCAGGGCGAGGTGCACGAGCTGAAGGGCGAGCTGGCCCGGATCCGGTCCGCGGCCGTGCTGCACCGCGAGATGGCACACAACCGGCTGGAGA

1 AGCGAACTGCACGGCCACCGGCTAGCGGACCGGCTGCGCAGCGTCGAGATCGAGCAGCGC

1 S E L H G H R L A D R L R S V E I E Q R

61 CGCCTTGCGGGCGCCAACTTCAACGTCAGCCGACAGATCGCCGGGCTGGACAAGCTGCAC

21 R L A G A N F N V S R Q I A G L D K L H

121 GGCTCGATGCTGGAGCTGCTCGAGGACGTGGAAGCGATACAGGGCAAGTTCGAGAAGACG

41 G S M L E L L E D V E A I Q G K F E K T

181 GTACCGGACATGCGGCGCGAAATCGCCAAGGTAGAGTTCAGCGTCGCGCAGGCCGCCTCC

61 V P D M R R E I A K V E F S V A Q A A S

241 GAGCAGGGGCTGGTGCGGGAGGAGGTGCACAACGCGGCCAAAAGCATCCAGGCGATGGCG

81 E Q G L V R E E V H N A A K S I Q A M A

301 GTCAGCGTGAGCGCGCTGCAGGAGGAGCGCGACACGGTCAAGCGGCTGCAGGGCGAGGTG

101 V S V S A L Q E E R D T V K R L Q G E V

361 CACGAGCTGAAGGGCGAGCTGGCCCGGATCCGGTCCGCGGCCGTGCTGCACCGCGAGATG

121 H E L K G E L A R I R S A A V L H R E M

421 GCACACAACCGGCTGGAG

141 A H N R L E

**>Vasa-Cas9-FREP1’** (478p PCR fragment from Vasa-Cas9 mosquito)

CTGAGCGAACTGCACGGCCACCGGCTAGCGGACCGGCTGCGCAGCGTCGAGATCGAGCAGCGCCGCCTTGCGGGCGCCAACTTCAACGTCAGCCGACAGATCGCCGGGCTGGACAAGCTGCACGGCTCGATGCTGGAGCTGCTCGAGGACGTGGAAGCGATACAGGGCAAGTTCGAGAAGACGGTACCGGACATGCGGCGCGAAATCGCCAAGGTAGAGTTCAGCGTCGCGCAGGCCGCCTCCGAGCAGGGGCTGGTGCGGGAGGAGGTGCACAACGCGGCCAAAAGCATCCAGGCGATGGCGGTCAGCGTGAGCGCGCTGCAGGAGGAGCGCGACACGGTCAAGCGGCTGCAGGGCGAGGTGCACGAGCTGAAGGGCGAGCTGGCCCGGATCCGGTCCGCGGCCGTGCTGCACCGCGAGATGGCACACAACCGGCTGGAGA

1 CTGAGCGAACTGCACGGCCACCGGCTAGCGGACCGGCTGCGCAGCGTCGAGATCGAGCAG

1 L S E L H G H R L A D R L R S V E I E Q

61 CGCCGCCTTGCGGGCGCCAACTTCAACGTCAGCCGACAGATCGCCGGGCTGGACAAGCTG

21 R R L A G A N F N V S R Q I A G L D K L

121 CACGGCTCGATGCTGGAGCTGCTCGAGGACGTGGAAGCGATACAGGGCAAGTTCGAGAAG

41 H G S M L E L L E D V E A I Q G K F E K

181 ACGGTACCGGACATGCGGCGCGAAATCGCCAAGGTAGAGTTCAGCGTCGCGCAGGCCGCC

61 T V P D M R R E I A K V E F S V A Q A A

241 TCCGAGCAGGGGCTGGTGCGGGAGGAGGTGCACAACGCGGCCAAAAGCATCCAGGCGATG

81 S E Q G L V R E E V H N A A K S I Q A M

301 GCGGTCAGCGTGAGCGCGCTGCAGGAGGAGCGCGACACGGTCAAGCGGCTGCAGGGCGAG

101 A V S V S A L Q E E R D T V K R L Q G E

361 GTGCACGAGCTGAAGGGCGAGCTGGCCCGGATCCGGTCCGCGGCCGTGCTGCACCGCGAG

121 V H E L K G E L A R I R S A A V L H R E

421 ATGGCACACAACCGGCTGGAGA

141 M A H N R L E

**>FREP1-gRNA-FREP1’ (**478bp PCR fragment from FREP1-gRNA expressing mosquito**)**

CTGAGCGAACTGCACGGCCACCGGCTAGCGGACCGGCTGCGCAGCGTCGAGATCGAGCAGCGCCGCCTTGCGGGCGCCAACTTCAACGTCAGCCGACAGATCGCCGGGCTGGACAAGCTGCACGGCTCGATGCTGGAGCTGCTCGAGGACGTGGAAGCGATACAGGGCAAGTTCGAGAAGACGGTACCGGACATGCGGCGCGAAATCGCCAAGGTAGAGTTCAGCGTCGCGCAGGCCGCCTCCGAGCAGGGGCTGGTGCGGGAGGAGGTGCACAACGCGGCCAAAAGCATCCAGGCGATGGCGGTCAGCGTGAGCGCGCTGCAGGAGGAGCGCGACACGGTCAAGCGGCTGCAGGGCGAGGTGCACGAGCTGAAGGGCGAGCTGGCCCGGATCCGGTCCGCGGCCGTGCTGCACCGCGAGATGGCACACAACCGGCTGGAGA

1 CTGAGCGAACTGCACGGCCACCGGCTAGCGGACCGGCTGCGCAGCGTCGAGATCGAGCAG

1 L S E L H G H R L A D R L R S V E I E Q

61 CGCCGCCTTGCGGGCGCCAACTTCAACGTCAGCCGACAGATCGCCGGGCTGGACAAGCTG

21 R R L A G A N F N V S R Q I A G L D K L

121 CACGGCTCGATGCTGGAGCTGCTCGAGGACGTGGAAGCGATACAGGGCAAGTTCGAGAAG

41 H G S M L E L L E D V E A I Q G K F E K

181 ACGGTACCGGACATGCGGCGCGAAATCGCCAAGGTAGAGTTCAGCGTCGCGCAGGCCGCC

61 T V P D M R R E I A K V E F S V A Q A A

241 TCCGAGCAGGGGCTGGTGCGGGAGGAGGTGCACAACGCGGCCAAAAGCATCCAGGCGATG

81 S E Q G L V R E E V H N A A K S I Q A M

301 GCGGTCAGCGTGAGCGCGCTGCAGGAGGAGCGCGACACGGTCAAGCGGCTGCAGGGCGAG

101 A V S V S A L Q E E R D T V K R L Q G E

361 GTGCACGAGCTGAAGGGCGAGCTGGCCCGGATCCGGTCCGCGGCCGTGCTGCACCGCGAG

121 V H E L K G E L A R I R S A A V L H R E

421 ATGGCACACAACCGGCTGGAGA

141 M A H N R L E

**>FREP1-KO (AgFREP1**^Δ^**^155^)** (in Fig. 1F lower panel: 323bp PCR fragment from FREP1-KO)

**
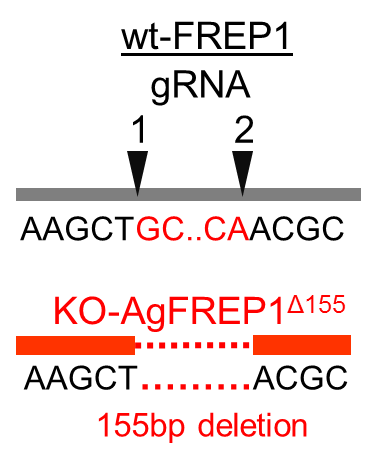
**

CTGAGCGAACTGCACGGCCACCGGCTAGCGGACCGGCTGCGCAGCGTCGAGATCGAGCAGCGCCGCCTTGCGGGCGCCAACTTCAACGTCAGCCGACAGATCGCCGGGCTGGACAAGCTAACGCGGCCAAAAGCATCCAGGCGATGGCGGTCAGCGTGAGCGCGCTGCAGGAGGAGCGCGACACGGTCAAGCGGCTGCAGGGCGAGGTGCACGAGCTGAAGGGCGAGCTGGCCCGGATCCGGTCCGCGGCCGTGCTGCACCGCGAGATGGCACACAACCGGCTGGAGA

1 CTGAGCGAACTGCACGGCCACCGGCTAGCGGACCGGCTGCGCAGCGTCGAGATCGAGCAG

1 L S E L H G H R L A D R L R S V E I E Q

61 CGCCGCCTTGCGGGCGCCAACTTCAACGTCAGCCGACAGATCGCCGGGCTGGACAAGCTA

21 R R L A G A N F N V S R Q I A G L D K L

121 ACGCGGCCAAAAGCATCCAGGCGATGGCGGTCAGCGTGAGCGCGCTGCAGGAGGAGCGCG

41 T R P K A S R R W R S A * A R C R R S A

181 ACACGGTCAAGCGGCTGCAGGGCGAGGTGCACGAGCTGAAGGGCGAGCTGGCCCGGATCC

61 T R S S G C R A R C T S * R A S W P G S

241 GGTCCGCGGCCGTGCTGCACCGCGAGATGGCACACAACCGGCTGGAGA

81 G P R P C C T A R W H T T G W R

**>FREP1-KO (AgFREP1**^Δ^**^1-gRNA1^)**

CTGAGCGAACTGCACGGCCACCGGCTAGCGGACCGGCTGCGCAGCGTCGAGATCGAGCAGCGCCGCCTTGCGGGCGCCAACTTCAACGTCAGCCGACAGATCGCCGGGCTGGACAAGCGCACGGCTCGATGCTGGAGCTGCTCGAGGACGTGGAAGCGATACAGGGCAAGTTCGAGAAGACGGTACCGGACATGCGGCGCGAAATCGCCAAGGTAGACTTCAGCGTCGCGCAGGCCGCCTCCGAGCAGGGGCTGGTGCGGGAGGAGGTGCACAACGCGGCCAAAAGCATCCGGGCGATGGCGGTCAGCGTGAGCGCGCTGCAGGAGGAGCGCGACGCGGTCGAGGGGCTGCGGGGCGAGGTGCACGAGCTGAAGGGCGAGCTGGCCCGGATCCGGTCCGCGGCCGTGCTGCACCGCGAGATGGCACACAACCGGCTGGAGA

1 CTGAGCGAACTGCACGGCCACCGGCTAGCGGACCGGCTGCGCAGCGTCGAGATCGAGCAG

1 L S E L H G H R L A D R L R S V E I E Q

61 CGCCGCCTTGCGGGCGCCAACTTCAACGTCAGCCGACAGATCGCCGGGCTGGACAAGCGC

21 R R L A G A N F N V S R Q I A G L D K R

121 ACGGCTCGATGCTGGAGCTGCTCGAGGACGTGGAAGCGATACAGGGCAAGTTCGAGAAGA

41 T A R C W S C S R T W K R Y R A S S R R

181 CGGTACCGGACATGCGGCGCGAAATCGCCAAGGTAGACTTCAGCGTCGCGCAGGCCGCCT

61 R Y R T C G A K S P R * T S A S R R P P

241 CCGAGCAGGGGCTGGTGCGGGAGGAGGTGCACAACGCGGCCAAAAGCATCCGGGCGATGG

81 P S R G W C G R R C T T R P K A S G R W

301 CGGTCAGCGTGAGCGCGCTGCAGGAGGAGCGCGACGCGGTCGAGGGGCTGCGGGGCGAGG

101 R S A * A R C R R S A T R S R G C G A R

361 TGCACGAGCTGAAGGGCGAGCTGGCCCGGATCCGGTCCGCGGCCGTGCTGCACCGCGAGA

121 C T S * R A S W P G S G P R P C C T A R

421 TGGCACACAACCGGCTGGAGA

141 W H T T G W R

Query: FREP1-KO (AgFREP1^Δ1^), Sbjct: Cas9-FREP1’

Query 1 CTGAGCGAACTGCACGGCCACCGGCTAGCGGACCGGCTGCGCAGCGTCGAGATCGAGCAG 60

||||||||||||||||||||||||||||||||||||||||||||||||||||||||||||

Sbjct 1 CTGAGCGAACTGCACGGCCACCGGCTAGCGGACCGGCTGCGCAGCGTCGAGATCGAGCAG 60

Query 61 CGCCGCCTTGCGGGCGCCAACTTCAACGTCAGCCGACAGATCGCCGGGCTGGACAAGC-G 119

|||||||||||||||||||||||||||||||||||||||||||||||||||||||||| |

Sbjct 61 CGCCGCCTTGCGGGCGCCAACTTCAACGTCAGCCGACAGATCGCCGGGCTGGACAAGCTG 120

Query 120 CACGGCTCGATGCTGGAGCTGCTCGAGGACGTGGAAGCGATACAGGGCAAGTTCGAGAAG 179

||||||||||||||||||||||||||||||||||||||||||||||||||||||||||||

Sbjct 121 CACGGCTCGATGCTGGAGCTGCTCGAGGACGTGGAAGCGATACAGGGCAAGTTCGAGAAG 180

Query 180 ACGGTACCGGACATGCGGCGCGAAATCGCCAAGGTAGACTTCAGCGTCGCGCAGGCCGCC 239

|||||||||||||||||||||||||||||||||||||| |||||||||||||||||||||

Sbjct 181 ACGGTACCGGACATGCGGCGCGAAATCGCCAAGGTAGAGTTCAGCGTCGCGCAGGCCGCC 240

Query 240 TCCGAGCAGGGGCTGGTGCGGGAGGAGGTGCACAACGCGGCCAAAAGCATCCGGGCGATG 299

|||||||||||||||||||||||||||||||||||||||||||||||||||| |||||||

Sbjct 241 TCCGAGCAGGGGCTGGTGCGGGAGGAGGTGCACAACGCGGCCAAAAGCATCCAGGCGATG 300

Query 300 GCGGTCAGCGTGAGCGCGCTGCAGGAGGAGCGCGACGCGGTCGAGGGGCTGCGGGGCGAG 359

|||||||||||||||||||||||||||||||||||| ||||| || |||||| |||||||

Sbjct 301 GCGGTCAGCGTGAGCGCGCTGCAGGAGGAGCGCGACACGGTCAAGCGGCTGCAGGGCGAG 360

Query 360 GTGCACGAGCTGAAGGGCGAGCTGGCCCGGATCCGGTCCGCGGCCGTGCTGCACCGCGAG 419

||||||||||||||||||||||||||||||||||||||||||||||||||||||||||||

Sbjct 361 GTGCACGAGCTGAAGGGCGAGCTGGCCCGGATCCGGTCCGCGGCCGTGCTGCACCGCGAG 420

Query 420 ATGGCACACAACCGGCTGGAGA 441

||||||||||||||||||||||

Sbjct 421 ATGGCACACAACCGGCTGGAGA 442

**>FREP1-KOs (Profile of other small deletion events)**

**
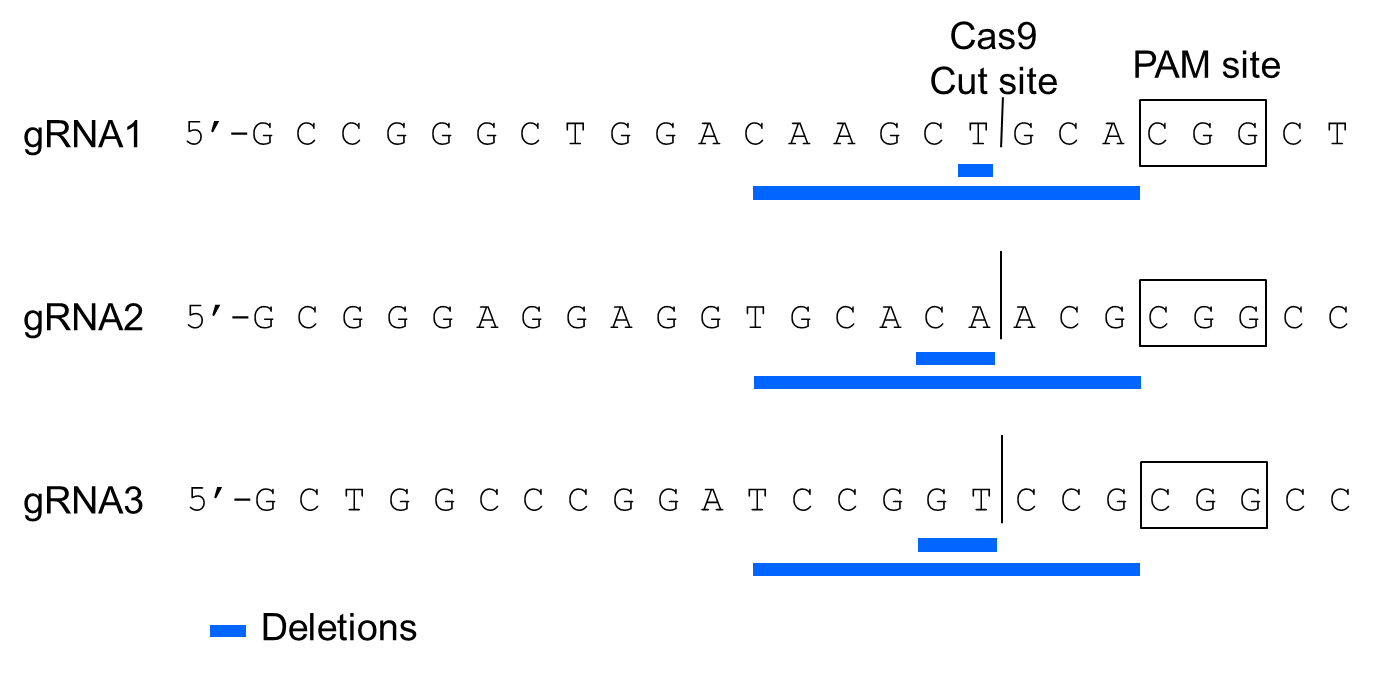
**
